# Biocomposites Based on PHBV and the Lignocellulosic Residue from Horchata Production

**DOI:** 10.3390/polym17070974

**Published:** 2025-04-03

**Authors:** Anita Patrón-Espá, María Eugenia Martín-Esparza, Amparo Chiralt, Chelo González-Martínez

**Affiliations:** Instituto Universitario de Ingeniería de Alimentos-FoodUPV, Universitat Politècnica de València, Camí de Vera s/n, 46022 València, Spain; eesparza@tal.upv.es (M.E.M.-E.); dchiralt@tal.upv.es (A.C.)

**Keywords:** compression molding, rigidity, total phenol content, tiger nut, antioxidant capacity

## Abstract

The use of agro-industrial residues in the development of packaging materials is a topic of interest from a sustainable perspective, as it promotes biodegradability, reduces production costs, and aligns with the concept of a circular economy. The aim of this work was to develop and characterize biodegradable composite films based on Poly 3-hydroxybutyrate-co-3-hydroxyvalerate (PHBV) and the tiger nut horchata solid residue (HSR) at different ratios. The obtained composites were evaluated as to their suitability as food active packaging materials in terms of microstructure, water content and solubility, mechanical, barrier and thermal properties, and total phenolic content and antioxidant capacity. The incorporation of HSR into the PHBV matrix led to more opaque, darker reddish films and promoted significant changes in their mechanical and barrier properties. Specifically, the composite films showed lower water vapor barrier capacity and reduced tensile strength (43–81% lower TS) and elongation at break (46–77% lower Ɛ values) while the rigidity increased or maintained when using up to 20% wt. of HSR. In contrast, the incorporation of the HSR provided the films with remarkable antioxidant capacity and effective light-blocking capacity, which could be of great interest for food preservation, as active packaging materials. The total phenol content of the composites increased in line with the increment of the HSR content, ranging from 9 to 34 mg GAE/100 g film.

## 1. Introduction

Biodegradable plastics currently represent roughly 0.25 percent of the over 400 million tonnes of plastic produced annually. This amount is expected to experience significant growth from 1.1 million tonnes in 2023 to approximately 4.6 million tonnes in 2028 [1], driven by the rising demand for more sustainable packaging materials and the emergence of more restrictive environmental policies.

The use of biodegradable materials coming from renewable sources to obtain active packaging for food could increase the shelf life of food while reducing waste, without the environmental impact of conventional plastics on marine and terrestrial ecosystems. One major hurdle limiting the expansion of the use of biodegradable plastics is the high production cost of biopolymers compared to fossil-based plastics, which limits their widespread adoption. Biodegradable plastics are directly obtained from biomass, synthesized from biobased materials, or biosynthesized by microorganisms. The production scale and processes made these polymers more expensive than those petroleum-based products used in conventional plastic production. Thus, the average cost of biodegradable plastics is around 2–5 times higher than traditional ones [2]. As the industry progresses and innovations continue, cost reductions may be realized, further increasing the appeal of these eco-friendly alternatives to conventional plastics.

An alternative to reduce the cost of these materials is to mix them with zero or low-cost materials such as waste generated by the agri-food industry. The food industry annually generates a large number of byproducts of low or no value, which are used for animal feed in the best scenario or discarded in landfills. For instance, the production of horchata, a vegetal refreshing beverage typical of Spain obtained from tiger nut tubers (*Cyperus esculentus* L.) generates a waste (HSR) that accounts for about 60 wt% of the original raw material [3]. This residue is susceptible to rapid fermentation, being used as organic mass for combustion, composting, or animal feeding, providing only minimal economic value [4]. The valorization of these wastes for the development of active food packaging could promote more sustainable materials while aligning with the principles of a circular economy. Its low cost, and richness in different polymeric compounds, such as starch, lignin, arabinoxylans, and cellulose [5], and polyphenols with antioxidant and antimicrobial activity, such as ferulic and *p*-coumaric acids or flavonoids [6], confers interesting and promising properties to this material for this purpose. Likewise, considering the non-cost of the horchata solid residue, the final cost of the packaging material would be reduced since the ratio of polymer is lower in the final material. Recently, several authors have studied the effect of incorporating different lignocellulosic waste materials such as rice husk, walnut shells, sugarcane bagasse, stem wheat, potato pulp, and spent coffee, among others into biodegradable polymer matrices to develop sustainable biopolymer-based composites and discover innovative ways to produce environmentally friendly materials [7]. However, no previous studies analyzed the potential of this byproduct to produce low-cost biodegradable composites.

Among the biodegradable materials with the greatest industrial projection, 3-hydroxybutyrate-co-3-hydroxyvalerate (PHBV) is attracting increasing interest due to its biobased and biodegradable nature in different environments (soil and marine), which can be used for food packaging due to its adequate mechanical and barrier properties [8,9]. In order to decrease the overall cost of PHBV-based materials while modulating their technical performance, PHBV can be combined with polymer-rich plant residues, such as HSR, to obtain biocomposites useful as food packaging materials.

The objective of the present study is to evaluate the ability of HSR to produce PHBV composite films by melt blending and compression molding, analyzing the effect of the different ratios of HSR particles on the microstructure of the PHBV composites, and their physical and functional properties. To this end, the water sorption and solubility of the films, mechanical, barrier properties, thermal behavior, and antioxidant capacity were analyzed. The proximate composition and microstructure of the HSR, its total phenolic content, radical scavenging capacity, and thermal stability were also determined for comparison purposes.

## 2. Materials and Methods

### 2.1. Raw Material

The vegetal residue (HSR) was provided by a local horchata producer Horchatería Rin (Valencia, Spain). This byproduct was dried at 50 ± 2 °C and milled in two cycles of 30 s at 10,200 rpm (Thermomix^®^ TM6, Vorwerk, Wuppertal, Germany). The powder was sieved (RP200N, CISA SIEVING TECHNOLOGIES, Barcelona, Spain) with a 300 µm mesh and stored for 2 weeks at 25 ± 1 °C and 0 relative humidity in a desiccator with P_2_O_5_ until its use.

Gallic acid, Folin–Ciocalteau reagent (2N), and 2,2-Diphenyl-1-picrylhydrazyl (DPPH) were obtained from Sigma-Aldrich (St. Louis, MO, USA). Sodium carbonate (NaCO_3_), phosphorus pentoxide (P_2_O_5_), and magnesium nitrate-6-hydrate (Mg(NO_3_)_2_) were supplied by Panreac Química, S.A. (Castellar del Vallès, Barcelona, Spain) and methanol by Applichem Inc. (Council Bluffs, IA, USA).

Poly(3-hydroxybutyrate-co-3-hydroxyvalerate) (PHBV) ENMAT Y1000P with 3% hydroxyvalerate was supplied by Helian Polymers B.V. (Belfeld, Holland). The PHBV was dried in a vacuum oven (VaciotermT, JP Selecta SA, Barcelona, Spain) at 60 °C for approximately 2 days, to remove the residual water content.

### 2.2. Characterization of HSR

The proximate composition of HSR was analyzed by the corresponding AOAC methods. Moisture (AOAC 934.06), protein (AOAC 920.152 using a conversion factor of 6.25), ash (AOAC 923.03), fiber (AOAC 991.43), and fat (AOAC 983.23) contents were determined in triplicate. Total starch was determined in triplicate using a Megazyme K-TSTA kit following the AOAC 996.11 method [10].

The microstructure of HSR particles was characterized using a high-resolution field emission scanning electron microscope (GeminiSEM 500, Zeiis, Oxford Instruments, Oxford, UK). Powdered samples were mounted on support stubs, coated with gold, and observed in a field emission scanning electron microscope (FESEM Ultra 55, Zeiss, Oxford Instruments, UK), using an acceleration voltage of 3 kV.

The total phenolic content (TPC) and the antioxidant capacity (EC_50_) of the residue were measured in triplicate according to the procedure described by Freitas et al. [11]. Methanol extraction of the phenols was carried out with a residue:methanol ratio of 1:15 (*w*/*v*) for 2 h at room temperature with agitation in darkness. The TPC was measured in the methanolic extract (filtered with 0.45 μm, Sigma-Aldrich, USA) through the Folin and Ciocalteu (FC) method following the methodology reported by Gomez-Contreras et al. [12]. The absorbance at 725 nm was measured using an Evolution 201 UV-Visible spectrophotometer (Thermo Scientific, Waltham, MA, USA) and expressed in mg Gallic Acid Equivalent (GAE) using a gallic acid standard curve (Abs = 0.099 × [mg GAE/L] + 0.043, r^2^ = 0.991). The values were expressed as mg GAE/100 g HSR.

The radical scavenging activity of the HRS methanolic extracts was also measured in triplicate, using the 2,2- Diphenyl-1-picrylhydrazyl (DPPH**∙**) free radical [13]. Briefly, different concentrations of the methanolic extract were added to the DPPH**∙** solution (6.22 × 10^−2^ mM) to reach a final volume of 4 mL and kept in the dark for 1.5 h. The decrease in absorbance at 25 °C was determined by using a spectrophotometer (Evolution 201 UV-Vis spectrophotometer, Thermo Scientific, EEUU) at 515 nm. The DPPH**∙** concentration was calculated from the calibration curve fitted by linear regression (Abs = 11.324 × [mM DPPH] − 0.0038, r^2^ = 0.9992). The radical scavenging activity was expressed through the EC_50_ value. This parameter was determined in the curves of the remaining DPPH**∙** (Equation (1)) vs. the ratio mg HSR/mg DPPH and corresponds to the amount of antioxidant per mass unit of radical required to decrease the initial DPPH**∙** concentration by 50%.

(1) $$\%\mathrm{DPPH}\cdot_{\mathrm{REM}}=\frac{[DPPH\cdot {]}_{t}}{[DPPH\cdot {]}_{0}}$$

A TGA analyzer (TGA 1 STARe System analyzer, Mettler-Toledo, Switzerland) was used to assess the thermal stability of the HRS under nitrogen flow (20 mL·min^−1^), by duplicate. Anhydrous samples of about 3–4 mg (pre-conditioned at 0% RH) were placed into aluminum pans and heated at 10 °C·min^−1^ from 25 to 700 °C. The thermogravimetric and their derivatives curves (DTG) were analyzed using STAR^e^ evaluation software (Mettler-Toledo, Switzerland) to obtain the initial degradation temperature (T_onset_), the temperature at the maximum degradation rate (T_peak_), and the residual mass at 600 °C.

### 2.3. Film Preparation

Five composite formulations were obtained by melt blending and compression molding, considering the HSR:PHBV ratios of 0:100, 10:90, 20:80, 30:70, and 40:60 (% *w*/*w*), where the percentage of HSR was used to identify the different formulations (PHBV, PHBV10, PHBV20, PHBV30, and PHBV40, respectively). To this aim, dry residue and PHBV were firstly melt-blended in an internal mixer (HAAKE^TM^ PolyLab^TM^ QC, Thermo Fisher Scientific, Dreieich, Germany) at 180 °C and 100 rpm for 4.5 min. Afterward, the blend was cold-grinded in a refrigerated batch mill (Model M20, IKA, Staufen, Germany). Finally, 4 g of the powder was placed onto Teflon sheets, preheated at 180 °C for 5 min in a hot-plate hydraulic press (Model LP20, Labtech Engineering, Bangkok, Thailand), then heated at 180 °C and 100 bar for 4 min and cooled down to 60 °C for 3 min. The obtained films were conditioned at 25 ± 1 °C and 53% relative humidity (RH) using Mg(NO_3_)_2_ oversaturated salt solution or at 0% RH, using P_2_O_5_, until further analysis.

### 2.4. Film Characterization

For cross-section microstructure analysis of the films, conditioned films at 0% RH were cryo-fractured by immersion in slush nitrogen, mounted on support stubs, coated with gold, and observed as described for HSR (Section 2.2).

The water content of the films was determined gravimetrically in quintuplicate in a vacuum oven (VaciotemT, JP Selecta SA, Barcelona, Spain) at 60 °C for 24 h. After that, the pre-dried films were placed in a desiccator with P_2_O_5_ at room temperature for total water removal until reaching constant weight.

The water vapor permeability (WVP) was determined in duplicate at 25 °C using the ASTM E-96-95 standard method [14], using a 100–53% RH gradient. For this purpose, 5 ml aliquots of distilled water were placed in Payne permeability cups (3.5 cm in diameter, Elcometer SPRL, Hermelle/Argenteau, Belgium), which were then inserted into a desiccator containing a saturated solution of Mg(NO_3_)_2_ (53% RH) and a fan placed on top. To determine the WVP of each sample, weight measurements were taken every 24 h for 1 week using an electronic balance (±0.00001 g, ME36S Sartorius, Data Weighing Systems, Inc., Wood Dale, IL, USA). The WVP was calculated based on the slope of the weight loss curves over time, taking into account the thickness of the film, which was measured using an electronic digital micrometer (Comecta S.A., Barcelona, Spain) in six random points.

Water solubility was determined in triplicate by immersing the samples in distilled water for 72 h using a film:water ratio (*w*/*w*) of 1:200. The wet samples were dried in a hot air convection oven at 60 °C (Dryterm, JP Selecta SA, Barcelona, Spain) for 48 h and, afterward, the samples were transferred to a desiccator with P_2_O_5_ for total water removal until reaching constant weight. Solubility (%) was calculated considering the mass of dry sample before and after the test and expressed as g water/100 g of film.

The mechanical properties of the films were determined with a universal texture analyzer (TA.XT plus, Stable Micro Systems, Haslemere, UK) in accordance with the ASTM D882 standard method [15]. Pre-conditioned 2.5 × 10 cm samples were stretched at 50 mm/min using A/TG tensile grips (initially separated by 50 mm) at room temperature. From the obtained strength–distance values, the stress vs. strain curves were calculated. From these curves, the mechanical parameters tensile strength (TS), percentage of elongation (Ɛ) at break, and elastic module (EM) were obtained. The analysis was performed on 8 replicates for each film.

The optical properties were determined in triplicate by measuring the surface reflectance spectra of the films from 400 to 700 nm using a spectrocolorimeter (CM-3600d, Minolta Co., Tokyo, Japan) on both black and white backgrounds. The transparency of films was assessed through the internal transmittance (T_i_) by applying the Kubelka–Munk theory for multiple scattering to the reflection spectra [16]. The internal transmittance (T_i_) was obtained by applying Equation (2), where R_0_ is the reflectance of the film on the black background, and parameters a and b were calculated by Equations (3) and (4), where R is the reflectance of the sample backed by a known reflectance (*R_g_*). CIE L*a*b* color coordinates were obtained from the reflectance of an infinitely thick layer of the material (*R∞*, Equation (6)) by considering D65 illuminant and 10° observer, according to Hutchings [16]. Chroma (C*_ab_) and hue (h*_ab_) and the color difference between the different composite films and the neat PHBV film (control) were also determined by equations (Equations (6) to (8)).

(2) $$T_{i}=\sqrt{{\left(a-R_{0}\right)}^{2}}-b^{2}$$

(3) $$a=\frac{1}{2}\cdot \left(R+\frac{R_{0}-R+R_{g}}{R_{o}\cdot R_{g}}\right)$$

(4) $$b={\left(a^{2}-1\right)}^{\frac{1}{2}}$$

(5) $$R_{\infty }=a-b$$

(6) $$∆E=\sqrt{{\left(∆L^{*}\right)}^{2}+{\left(∆a^{*}\right)}^{2}+{\left(∆b^{*}\right)}^{2}}$$

(7) $$h_{ab}^{*}=arctg\frac{b^{*}}{a^{*}}$$

(8) $$C_{ab}^{*}=\sqrt{a^{*2}+b^{*2}}$$

For the differential scanning calorimetry analysis (DSC823 STAR^e^ system, Mettler-Toledo, Inc., Greifensee, Switzerland), film samples (6–12 mg) were placed into aluminum-sealed pans, sealed, and subjected to two consecutive heating cycles. First, samples were heated from 25 °C to 200 °C at 10 °C·min^−1^, cooled down to −40 °C, and then, a second heating cycle was performed to 200 °C at 10 °C·min^−1^. The test was carried out in duplicate using an empty pan as reference. From the thermograms, the glass transition (T_g_) temperature, melting and crystallization temperatures (T_m_, T_c_), and melting enthalpy (ΔH_m_) were obtained. The percentage of crystallinity (X_c_) was calculated using 146 J/g PHBV for the melting enthalpy of totally crystallized PHBV [17]. A TGA of the film samples was performed in duplicate as described in Section 2.2. for the HSR.

The total phenol content and DPPH scavenging capacity of the films were also quantified as described in Section 2.2 for the HSR samples. To this end, the films were previously submitted to methanol extraction for 10 days at 400 rpm to favor the release of target compounds from the incorporated HSR.

### 2.5. Statistical Analysis

The statistical analysis of the results was made by applying the analysis of simple variance (ANOVA) using the software Statgraphics Centurión XVIII (StatgraphicsTechnologies, Inc., Rockville, MD, USA). Fisher’s least significant difference (LSD) with a confidence level of 95% was used to identify significant differences.

## 3. Results and Discussion

### 3.1. Properties of the HSR

**Figure 1:**
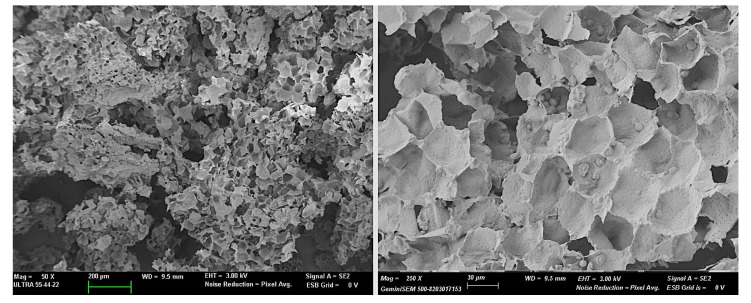
FESEM micrographs of the HSR particles at different magnifications.

The dry, ground and sieved HSR had a complex composition, with proteins (6.6 ± 0.5 wt. %), starch (26 ± 2 wt. %), fat (10.5 ± 0.7 wt. %), fiber (44 ± 2 wt. %), and ashes (1.3 ± 0.5 wt. %), combined with very little bound water at 1.9 ± 0.4 wt. %. These components are arranged in the particles of plant residue as shown in Figure 1, where the cell walls, mainly contributing to the total fiber content, and the remaining starch granules can be clearly observed. The cell walls mainly are composed of cellulose, hemicelluloses, and lignin [5].

The total phenol content of the HSR determined by the Folin–Ciocalteu method was 96 ± 18 mg/100 g of HSR, in the range of other plant residues such as beer bagasse (89–150 mg GAE/100 g dry sample) [12], which provide it with DPPH radical scavenging capacity, the EC_50_ values being 226 ± 18 mg HSR/mg DPPH. Nevertheless, TPC and EC_50_ values will be greatly affected by the extraction yield of the used method, which in turn, will be affected by the kind of solvent, particle:solvent ratio, temperature, and bonding degree of active compounds in the plant matrix [18]. Other authors obtain values of TPC ranging from 4.3–200 mg GAE/100 g dry HSR [18,19], depending on the extraction conditions. These extraction conditions also affected the phenolic profile of the extracts.

**Figure 2:**
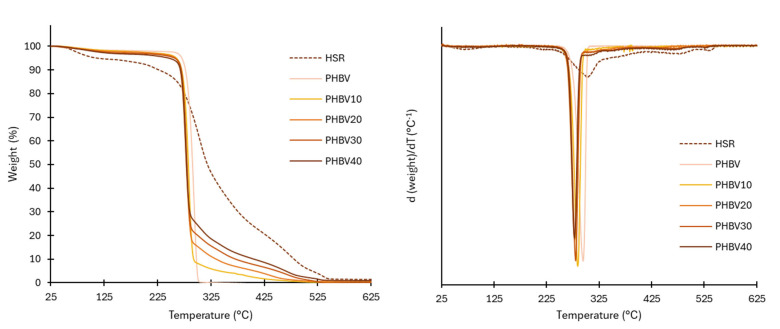
TGA and derivative curve (DTG) of the HSR, neat PHBV films, and composites.

The thermal stability of the HSR can be observed in Figure 2, which show, respectively, the TGA and DGTA curves. At least three thermal events could be detected: in the first step, small weight losses were observed up to nearly 200 °C, related to the loss of bound water and degradation of low molecular weight compounds, such as sugars, from the plant matrix. This first mass loss step at temperatures in the range of the film thermoprocessing temperature (180 °C) is important since these reactions will also occur in the HSR particles during the processing of the composites, leading to changes in the incorporated particles into the composites. The second wide degradation step, between 140 and 500 °C, corresponds with the overlapped degradation of polymeric constituents of HSR, defining the main degradation step (80% weight loss). Zhao et al. [20] reported about 20% cellulose and lignin and more than 15% of hemicellulose for tiger nut. Thus, the main degradation step can be in part attributed to the degradation of these constituents, which exhibited degradation at 150–350 °C for hemicellulose, 275–350 °C for cellulose, and 160–900 °C for lignin [21]. Starch (27 % in the HSR) also degrades between 250–350 °C [22]. The last degradation step above 400 °C can be attributed to the degradation of secondary products and residual lignin. During the processing of composites, no changes in the polymeric constituents are expected since the processing temperature did not exceed the onset degradation temperature of these components.

### 3.2. Characterization of Composite Films

#### 3.2.1. FESEM Microstructure

**Figure 3:**
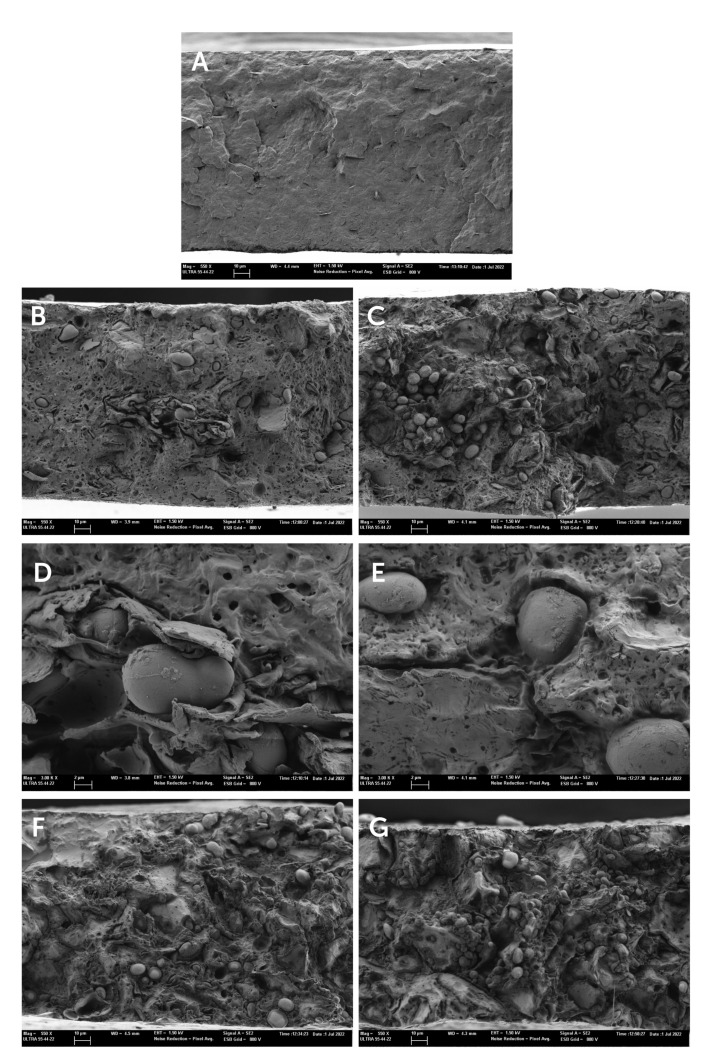
Field emission scanning electron microscopy (FESEM) images of the film cross-sections of (**A**) PHBV, (**B**,**C**) PHBV-10, (**D**,**E**) PHBV-20, (**F**) PHBV-30, (**G**) PHBV-40.

Microstructural composite morphology is one of the key parameters to support the observed properties of polymer composites [23]. The cross-section images of the composite films obtained by FESEM are shown in Figure 3.

Neat PHBV films showed the typical cross-section structure of a semicrystalline and homogenous material. The incorporation of the HSR resulted in a heterogeneous structure, this being more noticeable when the HSR concentration increased. In the continuous polymeric matrix, the presence of several dispersed phases (starch granules, lipid droplets, and fibrous particles) distributed throughout the matrix can be observed, fibers being better observed at the higher magnification in Figure 3D. It is noticeable that a large amount of unaltered starch granules was observed. This suggests that the temperatures used during the thermal processing of the films were not enough to cause their gelatinization, due to the absence of water or plasticizers such as glycerol, necessary to produce thermoplastic starch [24].

The presence of interfacial voids between starch granules and the polymer matrix (magnification in Figure 3E) was detected, pointing to a lack of interfacial adhesion between the hydrophilic starch granules and the polymer matrix. This lack of interfacial affinity would compromise the reinforcing effect of the granules and the mechanical performance of the composite films. An important ratio of small voids in the polymer matrix was also observed. These must be attributed to the dispersed oil phase of the HSR with around 10% fat, which evaporates under the high vacuum conditions of the microscope. Nevertheless, the formation of volatiles (such as CO_2_) during the film thermoprocessing could also occur as a consequence of the partial thermal degradation of some of the HSR components at the processing temperature (180 °C). In fact, TGA analysis of the HSR showed a mass loss step within the temperature range of 40–180 °C, attributable to the loss of bound water and caramelization of free sugars. Therefore, it is expected that these thermal phenomena also occur during the melt blending and thermocompression steps to obtain composite films. These microstructural features of the films will affect their functional properties, particularly, the mechanical and barrier properties, among others.

#### 3.2.2. Water Content, Water Solubility, Thickness, and Water Vapor Permeability

The values for the water content (x_w_), water solubility (S), thickness (t), and water vapor permeability (WVP) of the control (neat PHBV) and the composite films are shown in Table 1. The obtained values for the control film agreed with those found by other authors [25,26].

The incorporation of the HSR led to an increase in the film water content, being significantly higher (*p* < 0.05) when its mass fraction in the composite was greater than 20%, in agreement with the hydrophilic nature of the HSR material [27]. The water solubility of neat PHBV was significantly lower than that of composites (*p* < 0.05), in agreement with the solubilization of water-soluble compounds of the HSR. However, lower solubility was observed for composites with the highest HSR ratio, which suggests an increase in the interlocking capacity of the HSR compounds provoked by thermal treatment when these are present at a higher ratio in the composite. Conversely, composite films were significantly thicker (*p* < 0.05) when incorporating 20% or more HSR into the film matrix, probably due to the lower flowability of the blend during the thermocompression step, which depends on the blend viscosity and pressure applied [28].

The water vapor permeability of the composite films significantly increased (*p* < 0.05) when incorporating more than 10% HSR. This can be attributed to the hydrophilic nature of the residue, which makes the matrix more prone to water vapor transmission and to the low adhesion forces between dispersed particles and polymer. Thus, particles interrupt the continuity of the matrix, creating preferential channels for the diffusion of water molecules and gases [29]. Similar results have been found by other authors for PHBV-based films incorporating powdered rice straw, almond shell, rice husk, or seagrass [30,31].

#### 3.2.3. Mechanical and Optical Properties

Table 2 summarizes the mechanical parameters obtained for the control and composite films and the stress-strain curves for the different formulations are shown in Figure S1 (Supplementary File). The obtained values for the pure PHBV films were like those obtained by other authors working with unplasticized PHBV [32].

The mechanical properties of composite films can be affected by several factors such as the HSR volume fraction, aspect ratio of the particles, or the interfacial adhesion force between the different particles of the HSR and the polymer matrix [33]. In general, the mechanical resistance is more sensitive to the matrix properties and adhesion between residue components and the polymeric matrix while the material rigidity depends more on the residue concentration and its properties [34].

The composite films became less resistant to break (43–81% lower TS) and less stretchable (46–77% lower Ɛ values) than neat PHBV films, in agreement with other studies with PHBV biocomposites containing rice straw or wood fiber [35,36]. These effects were more noticeable as the residue content increased due to insufficient interfacial adhesion among components, which can lead to unsuitable stress transfer between fibers and matrix [37]. Furthermore, the large and unadhered particles such as the starch granules or fibers observed by FESEM, can act as obstacles in a polymeric matrix, leading to a premature failure. The EM values increased (*p* < 0.05) at low HSR concentrations (up to 10%) and then decreased when higher amounts were incorporated. At low HSR concentrations, the filler could also act as a nucleating agent, the polymer increasing the crystalline domains, thus leading to an increase in material rigidity. For larger concentrations, the HSR particles acting as imperfections and defects in the matrix are favored, thus reducing the mechanical performance of the material. Similar results were obtained by other authors working with different fillers and concentrations [38].

The incorporation of the HSR also promoted remarkable changes in the color properties of the films, which are shown in Table 3. Thus, composite films became more opaque (lower T_i_ values), leading to darker (lower L* values), more saturated in color (greater C*_ab_ values), and redder films (lower hue values) than neat PHBV (*p* < 0.05). The greater opacity of composite films could be beneficial in limiting the oxidation of foods sensitive to these spoilage mechanisms. These changes, evaluated throughout the color difference values (ΔE), were significantly greater (*p* < 0.05) when increasing the residue content.

#### 3.2.4. Thermal Behavior of the Films

Thermal gravimetric analysis (TGA) was used to measure the thermal stability of the obtained films. The TGA curves and their first derivatives (DTG), shown in Figure 2, are tighter than the corresponding curves obtained for HSR. The onset and endset degradation temperatures, and the temperature at the maximum degradation rate (T_peak_ in DTGA curves), are summarized in Table 4. As previously reported [8,20], neat PHBV films exhibited a single-stage thermal degradation, starting at around 270 °C, with a maximum degradation rate of 290 °C. This thermal degradation of PHBV follows a typical thermal degradation mechanism of polyesters in two stages [30]. The first step consists of a random chain cleavage through the removal of cis bonds, an intramolecular reaction that causes a drastic reduction in the molecular weight of the polymer and the formation of new acid terminals. Afterward, transesterification occurs between polymer chains, although this stage and/or reaction occurs to a lesser extent.

The thermal degradation of the composites started earlier (251–263 °C) than in the control films, in agreement with the lower thermal stability of the HSR. Nevertheless, this effect did not compromise the thermal processing of the films, which was carried out close to the polymer melting temperature (170 °C). The decrease in the onset temperature values rose as the films incorporated more HSR. The temperature at which the maximum degradation occurred (T_peak_) also decreased (*p* < 0.05) from 291 to 279 °C due to the overlapping of the degradation of the HSR polymeric components. The hydrophilic nature of the residue, which is able to bind water, could also contribute to a decrease in the PHBV degradation temperature, as this polymer has been proven to be very sensitive to water hydrolysis. This behavior was also observed by other authors for PHBV with hydrophilic fillers [30,39,40].

The residual mass percentage at 600 °C of the films (Table 4) was very low and increased in line with the residual mass of the HSR, associated with the ash content and other thermo-resistant components, such as lignin.

DSC analyses of the films were also performed to study the PHBV crystallization behavior in the composites. Table 5 summarizes the melting (first and second heating scans) and crystallization (cooling scan) temperatures for the different samples, as well as the corresponding melting enthalpy values, and Figure S2 (supplementary materials) shows the thermographs obtained for each formulation. The glass transition temperature (T_g_) was also determined in the first and second heating scans. In the first and second heating, neat PHBV films show a T_g_ value of around –4.3 °C, and T_m_ and T_c_ values of 172 °C and 120 °C for melting and crystallization, respectively, exhibiting a crystallinity degree (X_c_) of 42%, in agreement with that found by other authors [41,42]. When HSR was added, both T_m_ and T_c_ tended to decrease (maintaining the supercooling effect), while an increment in the polymer crystallization percentage was observed (*p* < 0.05), regardless of the amount of residue incorporated (in both the first and second scans). So, crystallization was favored by the presence of the HSR components, despite the lower crystal size (lower T_m_ values) formed by the blending effect. The increase in the ratio of crystalline domains affects the films’ mechanical response, enhancing the stiffness of the polymeric matrix, as commented on above for the samples incorporating the lowest HSR content (PHBV10). Nevertheless, the polymer matrix interruption by the HSR components provoked lower stiffness of the material when the HSR content rose.

#### 3.2.5. Antioxidant Capacity and Total Phenolic Content

The incorporation of HSR provides the PHBV composites with antioxidant activity evaluated through the total phenolic content (TPC) by the Folin–Ciocalteu method and the EC_50_ parameter for the DPPH radical. Table 6 shows these parameters, TPC and EC_50_ values, expressed as mg GAE/100 g film (TPC^1^) or mg GAE/100 g dry residue in the film (TPC^2^), and as mg residue/mg DPPH, respectively.

As commented on above, the TPC values in the HSR are in the range of those reported by other authors, being highly affected by the solvent and extraction method and the tiger nut variety [5,18,19]. The main identified phenolic compounds are phenolic acids and flavonoids, mostly remaining linked to the cellular walls of the plant tissue after the manufacture of horchata [18]. These TPC values are close to those reported for defatted beer bagasse (40–110 mg GAE/100 g) but lower than those found for rice straw (250–459 mg GAE/100 g) [11,43].

The TPC of the films, expressed per g of film (TPC^1^) increased in line with the increment in the HSR in the film. When TPC values were referred to g residue (TPC^2^), these values were also constant and similar to those found in the residue, thus indicating that phenolic compounds from the composite were efficiently extracted and no significant losses during the thermal processing of the films occurred.

The antioxidant capacities of the films, expressed in mg residue/mg DPPH, were remarkably higher (lower EC_50_) than that of HSR. Several authors have explained this effect as a consequence of the neoformation of Maillard compounds with noticeable antioxidant activity during the film thermoprocessing (melt blending and compression molding), such as melanoidins and HMF [44,45]. The high temperatures reached during the internal mixing of the blend and the film thermoforming process (180 °C) may promote the formation of these compounds, thus contributing to the total antioxidant capacity. Similar results have been reported for different plant residues submitted to severe thermal processes [12,46].

## 4. Conclusions

Low-cost PHBV composites could be obtained via melt blending by incorporating up to 40% of an under-utilized agri-food industrial waste such as the horchata production residue (HSR). Its incorporation promoted remarkable changes in the optical, vapor barrier capacity, and mechanical properties of the films, while their thermal stability remained unchanged, regardless of the residue concentration. Thus, composite films exhibited a dark reddish appearance, reduced water vapor barrier performance, and remarkable antioxidant capacity, with total phenol content ranging from 9 to 34 mg GAE/100 g of film. As concerns the mechanical properties, the resistance to break and stretchability of the composites were reduced up to 80% and 73%, respectively, by the presence of the residue particles. The film stiffness was either enhanced or maintained at low filler concentrations (up to 20%), likely due to the enhanced crystallinity of the polymer. These composites could be of interest for the development of low-cost, rigid packaging materials, such as trays, since the more expensive polymer can be replaced by up to 20% HSR, while the HSR provides the composites with potential antioxidant activity.

## Figures and Tables

**Table 1:** Water content (x_w_), solubility (S), thickness (t), and water vapor permeability (WVP) of neat PHBV and composite films. (mean ± standard deviation).

Film	x_w_ (g/100 g)	S (g/100 g Polymer)	t (µm)	WVP (g/Pa·s·m)·10^11^
PHBV	0.21 ± 0.08 ^a^	0.27 ± 0.13 ^a^	129 ± 14 ^a^	0.5 ± 0.2 ^a^
PHBV10	0.6 ± 0.1 ^a^	0.73 ± 0.02 ^c^	138 ± 14 ^a^	1.4 ± 0.2 ^a^
PHBV20	0.6 ± 0.2 ^a^	0.62 ± 0.02 ^c^	160 ± 22 ^b^	5 ± 2 ^b^
PHBV30	1.2 ± 0.2 ^b^	0.47 ± 0.04 ^b^	162 ± 14 ^b^	9 ± 2 ^c^
PHBV40	1.8 ± 0.5 ^c^	0.45 ± 0.07 ^b^	160 ± 20 ^b^	25 ± 3 ^d^

Different letters (a–d) within the same column indicate significant differences between samples (*p* < 0.05).

**Table 2:** Values of the Elastic Module (EM), Tensile Strength (TS), and Elongation at Break (Ɛ) of neat PHBV and composite films. (mean ± standard deviation).

**Film**	**EM (MPa)**	**TS (MPa)**	**Ɛ (%)**
PHBV	2922 ± 266 ^c^	32 ± 4 ^d^	1.32 ± 0.09 ^e^
PHBV10	3251 ± 512 ^d^	18 ± 4 ^c^	0.67 ± 0.12 ^d^
PHBV20	2726 ± 381 ^bc^	12 ± 2 ^b^	0.53 ± 0.07 ^c^
PHBV30	2534 ± 220 ^ab^	8 ± 2 ^a^	0.44 ± 0.07 ^b^
PHBV40	2198 ± 216 ^ab^	6.2 ± 0.6 ^a^	0.35 ± 0.06 ^a^

a–e: Different superscripts within the same column indicate significant differences among formulations (*p* < 0.05).

**Table 3:** Internal transmittance at 550 nm (Ti), lightness (L*), chroma (C*_ab_), hue (h*_ab_), and color difference (ΔE) for the neat PHBV and composite films. (Mean values ± standard deviation).

Film	T_i_ 550 nm	L*	C*_ab_	h*_ab_	ΔE
PHBV	0.64 ± 0.02 ^e^	71.5 ± 0.5 ^e^	18.9 ± 0.3 ^a^	77.5 ± 0.2 ^e^	-
PHBV10	0.38 ± 0.03 ^d^	55.0 ± 0.6 ^d^	26.7 ± 0.3 ^e^	66.3 ± 0.6 ^d^	18.7 ± 0.5 ^a^
PHBV20	0.28 ± 0.03 ^c^	46.9 ± 0.8 ^c^	25.4 ± 0.7 ^d^	60.84 ± 1.04 ^c^	26.1 ± 0.4 ^b^
PHBV30	0.23 ± 0.03 ^b^	42.5 ± 0.9 ^b^	23.9 ± 0.9 ^c^	56.5 ± 1.3 ^b^	30.4 ± 0.9 ^c^
PHBV40	0.15 ± 0.03 ^a^	39.± 2 ^ba^	22 ± 2 ^b^	53 ± 2 ^a^	33.2 ± 1.3 ^d^

a–e: Different superscripts within the same column indicate significant differences among formulations (*p* < 0.05).

**Table 4:** Thermal degradation parameters of the tiger nut residue, neat PHBV, and composites. (Mean values ± standard deviation).

Sample	T_on set_ (°C)	T_peak_ ^(1)^	T_end set_	Residual Mass _at 600°C_ (%)
Residue	140 ± 2 ^a (1)^	301 ± 2 ^c^	348 ± 3 ^d^	1.2
PHBV	269.9 ± 0.5 ^d^	291.1 ± 0.4 ^c^	312.4 ± 0.3 ^c^	-
PHBV10	263 ± 3 ^cd^	282 ± 3 ^bc^	303 ± 3 ^b^	0.35
PHBV20	259 ± 4 ^c^	278 ± 4 ^ab^	298 ± 4 ^ab^	0.38
PHBV30	259.6 ± 0.6 ^c^	278.9 ± 0.6 ^ab^	298.9 ± 0.6 ^ab^	0.29
PHBV40	251 ± 2 ^b^	278 ± 0.5 ^a^	298.4 ± 0.4 ^a^	0.89

Different letters (a–d) within the same column indicate significant differences between samples (*p* < 0.05). ^(1)^ Main degradation step.

**Table 5:** Thermal properties obtained from DSC analysis for PHBV and composite films: glass transition temperature (T_g_), melting temperature (T_m_), crystallization temperature (T_c_), melting enthalpy (ΔHm), and degree of crystallinity (X_c_). (Mean values ± standard deviation).

Film	1st Heating Scan				Cooling	2nd Heating Scan			
	T_g_ (°C)	T_m_ (°C)	ΔH_m_ (J/g polymer)	X_c_ (%)	T_c_ (°C)	T_g_ (°C)	T_m_ (°C)	ΔH_m_ (J/g polymer)	X_c_ (%)
PHBV	−4.3 ± 1.1 ^a^	172 ± 2 ^a^	61 ± 2 ^a^	42.2 ± 0.2 ^a^	120 ± 2 ^d^	−3.8 ± 0.6 ^a^	170 ± 2 ^a^	80 ± 11 ^a^	54.7 ± 0.8 ^a^
PHBV10	−3.6 ± 0.2 ^a^	169 ± 2 ^a^	73 ± 1 ^bc^	49.7 ± 0.3 ^bc^	117 ± 2 ^bc^	−2.9 ± 0.05 ^a^	169 ± 2 ^a^	101 ± 2 ^b^	69.3 ± 0.2 ^b^
PHBV20	−3.5 ± 0.1 ^a^	169 ± 2 ^a^	77 ± 5 ^c^	53.1 ± 0.3 ^c^	116 ± 3 ^bc^	−3.2 ± 0.01 ^a^	168 ± 2 ^a^	98 ± 7 ^b^	67.1 ± 0.5 ^b^
PHBV30	−3.5 ± 0.8 ^a^	170 ± 2 ^a^	67 ± 2 ^ab^	45.9 ± 0.2 ^ab^	116 ± 2 ^abc^	−3.7 ± 0.2 ^a^	168 ± 2 ^a^	93 ± 3 ^b^	63.9 ± 0.2 ^b^
PHBV40	−3.1 ± 0.3 ^a^	167 ± 3 ^a^	78 ± 4 ^c^	53.5 ± 0.3 ^c^	114 ± 2 ^a^	−2.9 ± 0.2 ^a^	166 ± 2 ^a^	100 ± 6 ^b^	68.5 ± 0.4 ^b^

a–c: Different superscripts within the same column indicate significant differences among formulations (*p* < 0.05).

**Table 6:** Total phenolic content (TPC) and antioxidant activity in terms of EC_50_ of the residue, neat PHBV, and composite films. (Mean values ± standard deviation).

Film or Residue	EC_50_ (mg dry residue/mg DPPH)	TPC^1^ (mg GAE/100 g film)	TPC^2^ (mg GAE/100 g dry residue)
Residue	67 ± 5 ^a^	-	96 ± 18 ^a^
PHBV10	6.6 ± 1.2 ^b^	9 ± 2 ^d^	90 ± 20 ^a^
PHBV20	5 ± 2 ^b^	15 ± 2 ^c^	77 ± 7 ^a^
PHBV30	6.3 ± 1.5 ^b^	27 ± 2 ^b^	90 ± 8 ^a^
PHBV40	6 ± 2 ^b^	34 ± 12 ^a^	94 ± 31 ^a^

a–d: Different superscripts within the same column indicate significant differences among formulations (*p* < 0.05).

## Data Availability

The data used to support the findings of this study can be made available by the corresponding author upon request.

## Supplementary Materials

The following supporting information can be downloaded at: https://www.mdpi.com/article/10.3390/polym17070974/s1, Figure S1: Typical stress–strain curves of the PHBV films incorporating or not incorporating HSR. Figure S2: DSC thermograms of PHBV films incorporating or not incorporating HSR
