# Supplementary material for: Biocomposites Based on PHBV and the Lignocellulosic Residue from Horchata Production
Source: Polymers (Basel). 2025 Apr 3;17(7):974. doi: 10.3390/polym17070974 (PMC11991278; doi:10.3390/polym17070974)
Supplement: Supplementary file 1 [file polymers-17-00974-s001.zip › polymers-3526255-supplementary.pdf]

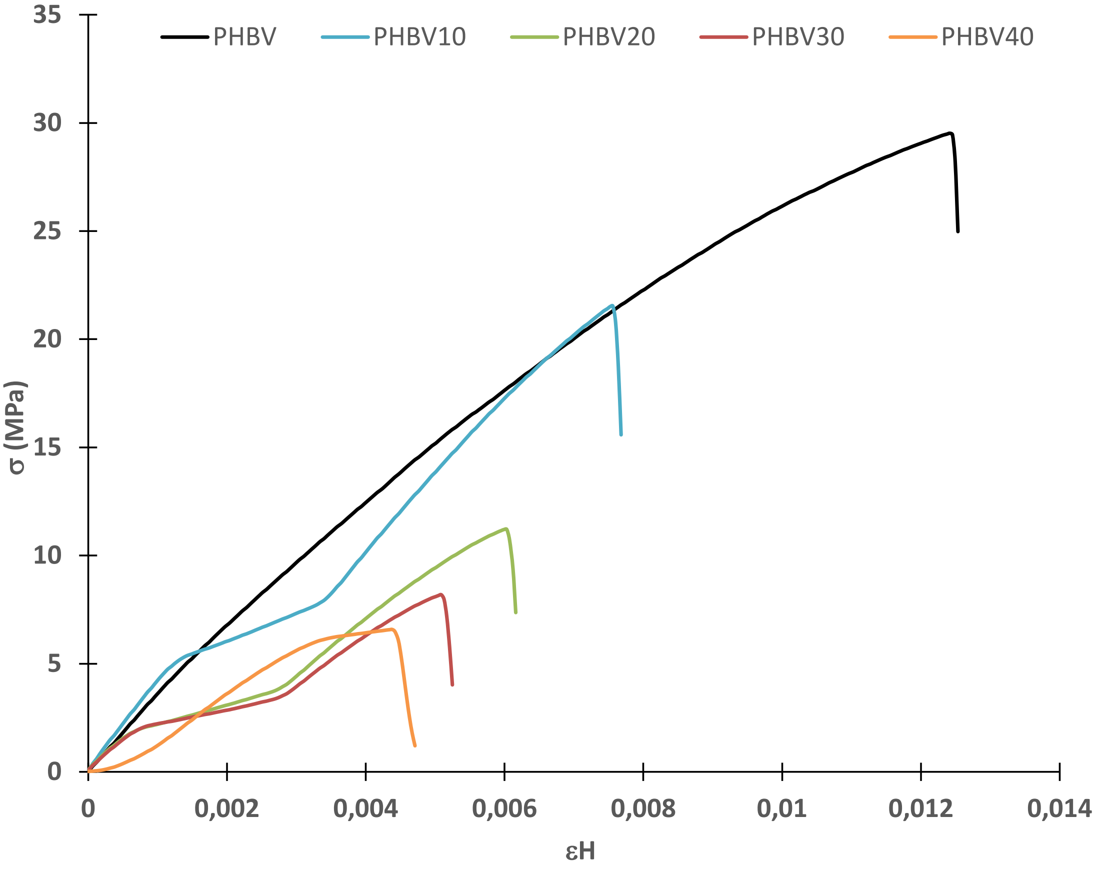

Figure S1. Typical stress–strain curves of the PHBV films incorporating or not incorporating HSR.

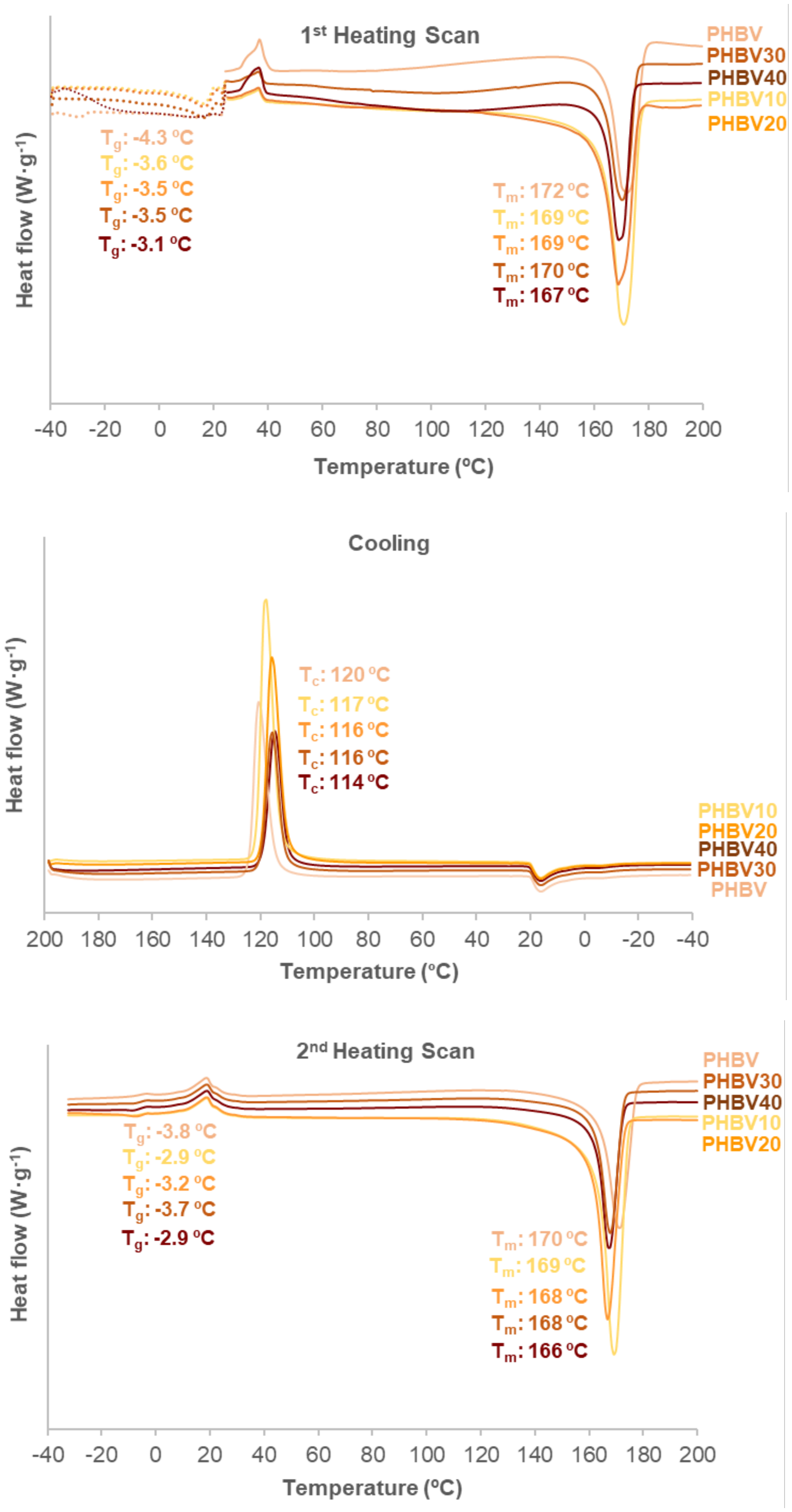

Figure S2. DSC thermograms of PHBV films incorporating or not incorporating HSR
